# Refining STEMI Prognosis: Expanding the Role of Noninvasive Cardiac Monitoring Beyond the GRACE Score

**DOI:** 10.1111/anec.70078

**Published:** 2025-04-07

**Authors:** Javeria Akhter, Javed Iqbal

**Affiliations:** ^1^ Lung Health Department, Community Health Directorate, Indus Hospital and Health Network Karachi Pakistan; ^2^ Nursing Department, Hamad Medical Corporation Doha Qatar

**Keywords:** clinical, electrophysiology, noninvasive techniques

Dear Editor,

We read with great interest the recent article by Xin et al. “Predictive Value of Noninvasive Cardiac Function Monitoring Combined with GRACE Score for Short‐Term Outcomes in Patients With ST‐Segment Elevation Myocardial Infarction” which provides valuable insights into the potential of noninvasive cardiac function monitoring (NCFM) to augment risk stratification in patients with ST‐segment elevation myocardial infarction (STEMI). The authors present a novel approach to improving prognostic accuracy for major adverse cardiovascular events (MACE) by integrating hemodynamic parameters with the established GRACE score (Xin et al. 2025). Although the study contributes implicitly to the field, certain aspects warrant further discussion.

First, the study successfully demonstrates that stroke volume (SV), cardiac output (CO), cardiac index (CI), contractility index (CTI), early diastolic filling ratio (EDFR), end‐diastolic volume (EDV), and systemic vascular resistance (SVR) are independent predictors of MACE. Moreover, the authors confirm that including SV and CTI into the GRACE score improves predictive performance. While this finding is promising, the study does not assess whether alternative combinations of hemodynamic parameters might offer even greater predictive accuracy. Considering the interaction of different cardiac function parameters, an exploratory analysis using machine‐learning techniques such as decision trees or neural networks could help investigate the most effective predictors of short‐term outcomes (Patel and Sengupta 2020).

Second, while the study effectively underscores the added predictive value of NCFM in combination with the GRACE score, it does not provide adequate discussion on the probability of integrating NCFM into clinical practice. Extensive implementation of noninvasive cardiac monitoring entails considerations such as availability, cost‐effectiveness, and user‐friendliness in different healthcare settings (Kim et al. 2019). Addressing these logistical concerns would enhance the study's clinical applicability and guide its possible adoption in routine patient management.

Third, the study does not consider probable confounding variables that may affect the predictive power of NCFM. Variables such as renal function, medication adherence, and previous cardiovascular interventions could affect both hemodynamic parameters and MACE outcomes (Chinwong et al. 2021; Hussain et al. 2023). Adjusting for these factors in a multivariate analysis would support the study's conclusions and provide more precise risk stratification.

Fourth, the study does not investigate the additional benefit of repeated NCFM measurements over time. Although the single‐timepoint evaluation at admission provides valuable prognostic information, dynamic changes in cardiac function parameters post‐STEMI may offer supplementary predictive value. Future research should assess whether serial NCFM measurements improve risk stratification beyond a single assessment.

Finally, while the study determines an improvement in predictive efficacy by altering the GRACE score, it does not compare this method against other recognized risk prediction models such as the TIMI risk score or the HEART score (Poldervaart et al. 2017). Given that these models are generally used for risk stratification in acute coronary syndromes, a comparative analysis would help explain the relative benefits of including hemodynamic indicators in current scoring systems and determine whether the proposed model provides a meaningful benefit over existing clinical practice.

In conclusion, Xin et al. present a groundbreaking study that improves STEMI risk stratification by integrating noninvasive hemodynamic parameters with the GRACE score. However, additional research is needed to explore alternative predictive models, measure the possibility of clinical implementation, adjust for further confounding factors, and assess the effectiveness of serial NCFM measurements. We commend the authors for their input and encourage constant investigation into refining risk prediction in STEMI patients.

## Author Contributions

The authors take full responsibility for this article.

## Ethics Statement

As this is a commentary on a published study and no new data were collected or analyzed, ethics approval was not required.

## Conflicts of Interest

The authors declare no conflicts of interest.

## Data Availability

The authors have nothing to report.

## References

[anec70078-bib-0001] Chinwong, S. , K. Doungsong , P. Channaina , A. Phrommintikul , and D. Chinwong . 2021. “Association Between Medication Adherence and Cardiovascular Outcomes Among Acute Coronary Syndrome Patients.” Research in Social and Administrative Pharmacy 17, no. 9: 1631–1635. 33455883 10.1016/j.sapharm.2021.01.003

[anec70078-bib-0002] Hussain, J. , H. Imsirovic , M. Canney , et al. 2023. “Impaired Renal Function and Major Cardiovascular Events in Young Adults.” Journal of the American College of Cardiology 82, no. 13: 1316–1327. 37730288 10.1016/j.jacc.2023.07.012

[anec70078-bib-0003] Kim, G. E. , S. Y. Kim , S. J. Kim , et al. 2019. “Accuracy and Efficacy of Impedance Cardiography as a Non‐Invasive Cardiac Function Monitor.” Yonsei Medical Journal 60, no. 8: 735–741. 31347328 10.3349/ymj.2019.60.8.735 PMC6660442

[anec70078-bib-0004] Patel, B. , and P. Sengupta . 2020. “Machine Learning for Predicting Cardiac Events: What Does the Future Hold?” Expert Review of Cardiovascular Therapy 18, no. 2: 77–84. 32066289 10.1080/14779072.2020.1732208 PMC7962010

[anec70078-bib-0005] Poldervaart, J. M. , M. Langedijk , B. E. Backus , et al. 2017. “Comparison of the GRACE, HEART and TIMI Score to Predict Major Adverse Cardiac Events in Chest Pain Patients at the Emergency Department.” International Journal of Cardiology 227, no. 15: 656–661. 27810290 10.1016/j.ijcard.2016.10.080

[anec70078-bib-0006] Xin, J. , Y. Liu , M. Ning , and C. Zhang . 2025. “Predictive Value of Noninvasive Cardiac Function Monitoring Combined With GRACE Score for Short‐Term Outcomes in Patients With ST‐Segment Elevation Myocardial Infarction.” Annals of Noninvasive Electrocardiology 30, no. 2: e70056. 40008483 10.1111/anec.70056 PMC11862889

